# Towards Sustainable Aquafeeds: Microalgal (*Nannochloropsis* sp. QH25) Co-Product Biomass Can Fully Replace Fishmeal in the Feeds for Rainbow Trout (*Oncorhynchus mykiss*)

**DOI:** 10.3390/foods14050781

**Published:** 2025-02-25

**Authors:** Pallab K. Sarker, Benjamin V. Schoffstall, Anne R. Kapuscinski, Brandi McKuin, Devin Fitzgerald, Connor Greenwood, Kira O’Shelski, Emily Noelle Pasion, Duncan Gwynne, Diego Gonzalez Orcajo, Sofie Andrade, Pablo Nocera, Angelo M. San Pablo

**Affiliations:** 1 Environmental Studies Department, University of California Santa Cruz, Santa Cruz, CA 95064, USA; 2 School of Engineering, University of California Merced, 5200 Lake Rd, Merced, CA 95343, USA

**Keywords:** aquafeed, fishmeal, microalgal co-product, defatted microalgae, aquaculture sustainability, rainbow trout, economic conversion ratio

## Abstract

Aquaculture, one of the world’s most rapidly growing food sectors, faces several concerns about its sustainability. A major concern is using fishmeal and fish oil from ocean-derived small pelagic fish (sardine, anchovy, etc.) in aquaculture feed. The aquafeed industry is seeking new sustainable ingredients to replace fish meal. This study focused on microalgal co-product, *Nannochloropsis* sp. QH25 co-product (leftover after oil extraction for nutraceuticals) is a novel aquafeed ingredient that can replace fishmeal in rainbow trout diets. A nutritional feeding experiment was conducted and compared fishmeal-containing rainbow trout diets with microalgal co-products that replaced fishmeal as follows: 0% replacement in reference diet (fishmeal, no microalgal co-product) and test diets with 33%, 66%, and 100% replacement of fishmeal using microalgal-product. Results showed the complete replacement diet yielded fish growth, feed conversion, and survival similar to the reference diet. Depositions of macronutrients, amino acids, fatty acids, macro minerals, and several trace elements in the filet were not significantly different across diets. Economic conversion ratio (ECR) analysis showed that the rainbow trout fed the 100% replacement diet had the lowest feed cost per kg of fish produced. Microalgal co-products can fully replace fishmeal in trout feed while maintaining fish performance, flesh composition, and cost-effectiveness.

## 1. Introduction

Aquaculture is one of the world’s fastest-growing food sectors, hitting a record of 20 million tons in 2020. Globally, nearly 50% of seafood consumed by humans is supplied by aquaculture, and this number is projected to increase [1,2,3].

While capture fisheries remain static or in decline, aquaculture is expected to fill the gap and drive consumer demand for fish products. Thus, the demand for sustainably produced and nutritionally complete aquafeeds increases, and there is a need to find alternative ingredients to fishmeal (FM) and fish oil (FO), for which aquaculture is the largest user [4]. Approximately 16 million of the 29 million tonnes of wild-caught forage fish global catch are currently rendered into aquaculture feed [5]. Since 2000, several developments have helped to reduce the aquaculture industry’s dependence on wild fish resources, although FM and FO remain critical as feed ingredients [6]. The use of FM and FO in aquafeeds has caused several sustainability concerns [3,7,8,9,10,11,12,13,14,15,16].

Creating innovations in feeds requires novel ingredients [17]. This is reflected in the current feed formulations used in the commercial production of rainbow trout (*Oncorhynchus mykiss*) because the calculated FIFO ratio has been significantly reduced due to the increased use of non-marine proteins and oils [18,19,20]. According to [20], the corresponding FIFO for the overall production of trout and salmon is 0.7 and 1.8, respectively. However, aquaculture experts expect that FM prices will rise steeply, and supplies will be insufficient to meet growing aquafeed demands and thus constrain aquaculture growth [21,22]. The continued growth of aquaculture will require the availability of cost-effective and nutritionally comparable alternatives to FM. In the past decade, several protein meals, including terrestrial protein meals (such as soy products, corn, and rapeseed) and animal by-products (poultry, feather, and blood meal) have been tested as alternative or supplementary protein sources for rainbow trout and Atlantic salmon (*Salmo salar*) in aquafeed formulations [23,24,25,26,27]. Most of them are promising alternatives to fishmeal due to their high protein content, relatively low price, and broad availability. However, most of the conventional proteins such as crop meals lack essential amino acids (i.e., lysine, methionine, threonine, and tryptophan) [28,29], important fatty acid fraction for human health, including long-chain omega-3s (n-3s), eicosapentaenoic acid (EPA) and docosahexaenoic acid (DHA) [7,30], and they have elevated antinutrients that are passed on to the consumers. In addition to the nutritional limitations, the competition from human food and livestock diets limits its further application in trout feed. To expand the production of aquafeeds, it is necessary to incorporate sustainable substitutesforFM, which have nutritional benefits and characteristics that support the health of both fish and seafood consumers [31,32].

The marine microalga, *Nannochloropsis* sp. product shows promise as a potential substitute for FM in feeds for farmed rainbow trout and Atlantic salmon because these species possess high protein contents, long chained polyunsaturated fatty acids (LC-PUFA), or both [7,33,34,35,36]. Limited studies have been conducted on the effects of incorporating *Nannochloropsis* sp. in the diets of trout and Atlantic salmon. Previous research also showed that *Nannochloropsis* biomass is a digestible source of protein and EPA for salmonids and could potentially be used as a protein ingredient in aquafeed formulations. Several nutritional feeding experiments have been conducted with rainbow trout and Atlantic salmon, and they have shown that up to 10% of *Nannochloropsis* sp. biomass can be included in the feed without any adverse effects on growth [35,37].

One of our recent studies showed that replacing 100% of FM and FO by combining two marine microlgae, *Nannochloropsis oculata* defatted biomass and *Schizochytrium* sp., in a Nile tilapia (*Oreochromis niloticus*) diet achieved higher final weight, weight gain, percent weight gain, specific growth rate, and protein efficiency ratio values comparable to the reference diet containing FM and FO [7]. Biomass from marine microalgae currently has a lower environmental impact than ingredients from crops grown in soil. They need less land, and can produce at least two times more biomass and significantly more oil and protein per hectare than soy [38,39]. Additionally, marine microalgae can be cultivated with wastewater or saline water instead of freshwater and they can thrive in non-arable sites, which avoids competition with agriculture for human food resources [32].

In this study, the feasibility of fully replacing FM ingredients in rainbow trout aquafeed by using protein-rich defatted biomass of *Nannochloropsis* sp. QH25 (algal coproduct (ac)), left over from industrial production for human nutraceuticals, into value-added aquafeed ingredients was tested [7,35,40].

The experiment aimed to achieve the following objectives:

1. Test different levels of (ac) inclusion (0%, 33%, 66%, 100%) to replace a low fish meal-containing commercial rainbow trout diet through a nutritional feeding trial.
2. Estimate the cost of the formulated feed and the economic conversion ratio (ECR) of the three different levels of fish meal replacing diet and the reference diet using the Cruz Aquafeed Sustainability Tool (CAST).

## 2. Materials and Methods

We designed a 64-day growth experiment replacing FM with (*ac*) QH25 co-product at different replacement levels. This experiment was carried out at the Ecological Aquaculture Lab at the Center for Agroecology at the University of California Santa Cruz, USA, under a protocol approved by the Institutional Animal Care and Use Committee (IACUC).

### 2.1. Diet Formulation and Nutritional Feeding Experiment

The nutritional feeding trial incorporated defatted (*ac*) to replace three different percentages of FM in three experimental diets. These three diets were compared to a reference diet where the FMFO composition was equivalent to commercial rainbow trout feed levels. All diets were iso-nitrogenous and iso-energetic and were formulated to ensure that they meet/satisfy the known nutritional requirements of rainbow trout [41,42]. Microalgal diets used (*ac*) to replace 33% (33N), 66% (66N), and 100% (100N) of FM in a low fishmeal (7.5%) reference diet (Table 1).

Dried defatted (*ac*) was obtained from Qualitas Health Inc. The defatted co-product used in this experiment consists of (*ac*) cells left over after using a non-toxic GRAS solvent (generally regarded as safe by the FDA), to extract oils for a human nutraceutical [40]. Menhaden FO from Double Liquid Feed Service, Inc., Danville, IL, USA. The (*ac*) comprised 4.1 g, 7.4 g, and 10 g per 100 g of the 33N, 66N, and 100N diets respectively. It is important to mention that the diets utilized in this experiment were balanced feed for rainbow trout, as per our earlier studies [42]. The reference and experimental diets included other protein sources such as feather meal (15%), blood meal (7%), corn gluten meal (20%), and soy protein concentrate (20%). Feather meal (80 g/100 g crude protein, 1 g/100 lipid; 6 g/100 ash), blood meal (82 g/100 g crude protein; 1.8 g/100 lipid; 6.4 g/100 ash), corn gluten meal (60.43 g/100 g crude protein; 3 g/100 lipid; 1.6 g/100 ash), and soy protein concentrate (55.4 g/100 g crude protein; 0.5 g/100 lipid; 6.4 g/100 ash) were commercially available ingredients were used in the feeds. The fishmeal used in the diets contained (63.8 g/100 g crude protein; 11.6 g/100 lipid; 0.5 g/100 ash. Correspondingly, Tables S1–S3 report the proximate and amino acid composition, fatty acid composition, and macrominerals and trace element compositions of the dried defatted (*ac*). Tables S4 and S5, respectively, describe fatty acid composition, and macrominerals and trace element composition of the three experimental diets and reference diet.

Each diet contained Yttrium oxide (Y_2_O_3_), an indigestible marker sourced from Thermo Scientific, Waltham, MA, USA, in the basal diet at a rate of 1.0%. To create the feed, the micro-ingredients were mixed first, followed by macro-ingredients, which were slowly added and thoroughly mixed into the feed to maintain a homogenous texture. Diets were manufactured at the Kapuscinski-Sarker Lab space in Natural Sciences II (University of California, Santa Cruz, CA, USA) using a single-screw extruder (TT-100 tabletop lab scale extruder from Akron Tool and Die, Akron, OH, USA). During extrusion, the diet was exposed to an average target temperature in the barrels at 90 °C and passed through the extruder for 18 s exposure. All diets were top coated with FO using a 1 − ¼ Cu Ft. rotating mixer (Harbor Freight, Calabasas, CA, USA) (and 24-mm mercury pressure. After mixing feed for 15 min, the feed was dried overnight to 13–18% moisture content in a fume hood (Table 1). The pellets were then sieved and stored at −20 °C. Initially, the pellets were 2.0 mm in diameter, but as the fish grew, we increased the pellet size to 4.0 mm to meet the nutritional demands of the larger fish.

### 2.2. Fish Husbandry and Experimental Design

The growth study used recirculating aquaculture systems at the University of California, Santa Cruz, CA, USA, consisting of sixteen 757-L tanks filled with dechlorinated city water, each containing 33 juvenile rainbow trout with an average weight of 40.5 ± 7 g each. The rainbow trout used in the study were purchased from Thomas Fish Company located in Anderson, CA, USA. Following placement in the tanks, the fish were allowed to acclimatize for seven days. After the acclimation period, the fish were randomly assigned to one of the treatments which consisted of three experimental diets and a reference diet. All of the fish were fed until apparent satiation twice a day, in the morning and afternoon, six days a week, for 64 days [42,43]. A total of 16 tanks were used, with four replicate tanks per diet. Water chemistry data was collected for each tank to maintain the recommended conditions for rainbow trout. Dissolved oxygen, dissolved oxygen saturation, temperature, and pH were sampled daily using a handheld 1020Pro multiparameter (YSI, Yellow Springs, OH, USA) meter to keep dissolved oxygen at 8.7 mg/L, the water temperature at 15.4 °C, and pH at 8.6. Levels of ammonia, nitrite, nitrate, and the alkalinity of the water were sampled weekly using a benchtop 9500 spectrophotometer (YSI, Yellow Springs, OH, USA) to maintain total ammonia nitrogen at 0.2 mg/L, nitrite nitrogen at 0.1 mg/L, and nitrate nitrogen at 26.8 at mg/L.

Fish fecal samples were collected daily before feeding using a radial flow settler. Intact fecal matter was collected at the bottom of the system by installing a radial flow settler between the culture tank outflow and the sump tank inflow. Uneaten feed pellets were siphoned from the radial flow settler to prevent contamination. Intact solid fecal matter was gently removed from a separate collection bin using pipettes. The fecal matter was placed in a 50 mL BD Falcon^TM^ conical tube (Becton, Dickinsin, and Company, Franklin Lakes, NJ, USA) and was allowed to settle at the bottom of the tube. Once the fecal matter settled at the bottom of the tube, the supernatant water at the top of the tube was removed using a pipette. The fecal matter was then frozen at −20 °C. Fecal samples from every collection were pooled from every tank used during the experiment. At the end of the experiment, it was lyophilized, finely ground, and stored samples at −20 °C for nutrient analysis. Five fish (3 for whole body and 2 for filet) were removed for initial growth data at the beginning of the experiment. Another five fish (3 for whole body and 2 for filet) were removed from each tank during the final sampling event to serve as replicates for the dietary growth and nutrient analysis treatments.

### 2.3. Growth Calculations

The final weight, weight gain, weight gain percentage, feed conversion ratio (FCR), specific growth rate (SGR), protein efficiency ratio (PER), and survival rate for each dietary treatment were quantified using the following equations:

Weight gain (g) = (Whole body_final wt._ − Whole body_initial wt._) (1)

Weight gain percent = [(Whole body_final wt._ − Whole body_initial wt._)/Whole body _initial wt._] × 100 (2)

FCR = Feed intake (as fed basis)/Weight gain(3)

SGR = 100 × (ln final wet weight (g) − ln initial wet weight (g))/Time (days)(4)

PER = weight gain (g)/protein fed (g)(5)

Survival rate = (Final number of fish/Initial number of fish) × 100(6)

### 2.4. Macronutrients and Trace Element Analysis

The chemical analyses used the standard methods described in our recent articles [12,42]. The nutrient composition of the samples ware determined after homogenizing the samples by grinding and then freezing the ingredients, diets, feces, and whole body fish to −20 °C. The samples were then prepared for ICP-OES analysis using the EPA Method 3050b [44] acid digestion method. The digested material was analyzed for elemental composition by ICP-OES (iCap 7400 radial view ICP-OES, Thermo Fisher Scientifc USA), Optical Emission Spectroscopy was conducted at UC Santa Cruz Plasma Analytical Laboratory, RRID: SCR_021925.

Three sample types (microalgal co-product, diets, and feces) were sent to New Jersey Feed Laboratory, Inc. (Ewing, NJ, USA) for analysis. The analyses included moisture (Association of Official Analytical Chemists, AOAC, 1995, method 930.15), crude protein (AOAC 990.03), lipid (AOAC 920.39), ash (AOAC 942.05), crude fiber (AOAC 1978.10), energy (automated oxygen bomb calorimeter), amino acids (high-performance liquid chromatography analysis, via AOAC methods 994.12, 985.28, 988.15, and 994.12), fatty acids (fatty acid methyl ester analysis, via AOAC method 963.22.), and yttrium oxide in feed and feces according to methods described by Cho, Slinger, and Bayley (1982) [45].

Once the concentration of the nutrients and yttrium levels of the diets and feces were determined, the apparent digestibility coefficient (ADC) of whole body proximate, amino acids, and essential amino acids was calculated.

The following equation was used to determine the ADC for the four diets [45]:

ADC = [1 − ((%Nutrients _feces_/%Nutrients _feed_) × (%Y_2_O_3 feed_/%Y_2_O_3 feces_)) × 100] (7)

### 2.5. Economic Conversion Ratio

The Cruz Aquafeed Sustainability Tool (CAST, https://cast.sites.ucsc.edu/ accessed on 3 June 2024) application was used to calculate the economic conversion ratio (ECR). CAST used the market prices from the CAST database to estimate ingredient prices [46]. In CAST we used the option to apply the experimental FCRs of this study instead of the default that is calculated by an algorithm in the software. The market prices of the experimental diets from the CAST database were obtained from a variety of sources (Table S6). The median values and 95% confidence intervals of the market prices in CAST were calculated from non-parametric bootstraps in RSTUDIO (v.1.2.5033) based on 10,000 replicates using the adjusted bootstrap percentile method.

The fish production cost was estimated as an ECR using the equation of [42]:

ECR ($/kg fish) = FCR ((kg diet fed)/(kg weight gain)) × price of diet (USD$/kg diet)(8)

where ECR is the economic conversion ratio, and FCR is the feed conversion ratio.

### 2.6. Statistical Analysis

A one-way ANOVA function using an IBM Statistical Package called Statistical Product and Service Solutions (SPSS) program for Windows (v. 27.0, Armonk, NY, USA) was used when determining the level significance between treatment groups. With the one-way ANOVA test, it was included a homogeneity of variance test to ensure the assumption of normality and the assumption of homogeneity of variance were met. It was included a Tukey post hoc test, with a 95% confidence interval to determine similarities and differences between treatments (*p* < 0.05).

## 3. Results

### 3.1. Growth and Feed Performance

Table 2 summarizes the results of the 64-day growth experiment. This experiment was conducted to determine the effects of replacing different percentages of dietary FM with (*ac*). Results showed that the rainbow trout fed reference diet had similar final weight, weight gain, percent weight gain, feed conversion ratio (FCR), protein efficiency ratio (PER), feed intake and specific growth rate (SGR) as all test diets replacing FM with the (*ac*), even when it wholly replaced FM (100N).

All fish appeared healthy and no signs of disease were observed throughout the study.

Additionally, no significant differences (*p* > 0.05) were found in the whole body proximate composition (Table S7) were found across the dietary treatments. Whole body composition analysis included moisture, ash, total lipid, and crude protein. The lipid content ranged between 34.66% and 35.19%, and protein contents ranged between 55.83% and 56.2% across the four diet treatments.

### 3.2. Filet Proximate, Amino Acids, and Fatty Acid Profiles

Filet proximate composition was compared across all treatments (Table S8). There were no significant differences (*p* > 0.05) in protein, fat, fiber, and ash between treatments. The lipid content ranged between 18.14% and 19.74%, and protein contents ranged between 73.38% and 75.51% across the four diet treatments. The reference diet yielded the highest protein content and the 33N diet had the highest lipid content.

Table 3 shows no significant differences (*p* > 0.05) for filet essential amino acid content among the four treatments. Filets of fish fed the reference diet had slightly higher values of methionine, lysine, phenylalanine, leucine, isoleucine, threonine and valine. The ranges of filet amino acid levels were 6.39–6.68% for lysine, 2.01–2.14% for methionine, and 0.47–0.61% for tryptophan.

Amounts of lipid and major omega-3 (n-3) and n-6 polyunsaturated fatty acid (PUFA) in the trout filet are reported in Table 4. The major fatty acid fractions in the filet did not differ between diets.

Fatty acid profiles of fish filets were compared among the reference diet and three microalgal co-product diets, as shown in Table 5. Results indicated that concentrations of certain fatty acids, including n-3 PUFA, n-6 PUFA, EPA, DHA, total PUFA, and total n-3 LC PUFA, were not significantly different (*p* > 0.05) among the diets. Additionally, there were no significant differences in the total saturated fatty acid (SFA), SFA fractions, total MUFA, and MUFA fractions across the different diets.

### 3.3. Filet Macro and Micro Minerals Composition

No significant differences (*p* > 0.05) were found in filet macro minerals composition among the four diets (Table 6). The reference and 33N filet mineral composition showed slightly higher amounts of calcium than the 66N and 100N filets. However, the differences were insignificant (*p* > 0.05). Among the trace elements detected, only lead showed a significant difference between the reference diet and 100N diet, while the rest showed no significant differences. The detected trace elements were iron, zinc, mercury, lead, and arsenic. However, arsenic was only detected in the 66N filet (0.002 mg kg^−1^), while the other trace elements were comparable across all diets (0.001 mg kg^−1^). Manganese, selenium, boron, aluminum, or molybdenum were not detected among the treatments due to the filets not containing these elements or the levels being below the detection level of the instrument.

### 3.4. Economic Conversion Ratio (ECR)

Table 7 shows the highest replacement diet (100N) had the lowest formulated feed cost ($0.88/kg feed) and ECR ($0.86/kg rainbow trout). However, the formulated feed costs and ECRs (the feed cost per kg fish produced) did not differ significantly (*p* > 0.05) among diets. The 33N diet had a less expensive feed formulation than the reference diet but had the highest FCR (1.01) and yielded a higher ECR (0.94), although these values did not differ significantly among diets.

### 3.5. Nutrient Digestibility

Table 8 reports the ADCs of the test diets’ macronutrients, energy, amino acids, and fatty acids. The ADC of dry matter in the 33N diet (99.52%) was significantly higher (*p* < 0.05) compared to the other diets (99.09–99.25%). Also, the ADC of crude fiber was significantly lower in the reference diet (99.13%) compared to the experimental microalgal co-product diets (99.39–99.43%). Tehe ADC of energy was significantly lower (*p* < 0.05) in the reference diet (97.97%) and the ADC of energy was significantly higher (*p* < 0.05) in the 100N diet (99.98%). The ADC levels of crude protein (99.26–99.29%), lipids (98.89–99.13%) and ash (86.23–84.65%) showed no significant differences (*p* > 0.05). The essential amino acids ADCs were higher but not significantly in the 100N diet compared to the reference diet except for tryptophan (Reference—99.57% and 100N—99.50%). The ADCs of total SFA (66.23–73.59%), total PUFA (90–90.53%), 22:5n-3 DHA (97.64–98.04%), total n-3 PUFA (95.25–95.53) and total n-6 PUFA (94.65–95.15%) were significantly higher in the microalgal co-product diets compared to the reference diet (total SFA—29.11%, total PUFA—79.73%, 22:5n-3 DHA—95.64%, total n-3 PUFA—89.92% and total n-6 PUFA—90.82%). The ADCs of total MUFA (37.9–60.27%) and 20:5n-3 EPA (96.64–98.27%) did not differ significantly (*p* > 0.05) between diets, although the reference diet had the highest total MUFA ADC and the 33N had the highest 20:5n-3 EPA ADC.

## 4. Discussion

To our knowledge, this study is the first evidence that rainbow trout can attain similar growth, FCR, muscle amino and fatty acids, minerals, and survival rates when given a microalgal co-product diet that replaces FM entirely yet remains cost-effective, compared to a reference FM diet. This study clearly shows that the high (100N) inclusion of (*ac*) microalgae co-product in rainbow trout diets is comparable to FM based diet, with no significant differences in growth metrics, fatty acid composition, and lower economic conversion ratio.

### 4.1. Effects of Microalgal Co-Product Feeds on Growth Performance

All the treatment groups did not differ significantly in feed intake, growth, weight gain, SGR, FCR, PER. Other studies, as well as one of our previous studies, have found contrasting results when *Nannochloropsis* sp. is used as a substitute for FM and FO in the diet of rainbow trout [7,47]. Our previous study reported that the rainbow trout fed the microalgae-based feed had significantly lower growth due to reduced feed intake [7]. To address this, feeding stimulants such as taurine and lecithin were added to all of the diets to improve palatability and growth performance. When taurine was added to the microalgal feed, there was an increased feed intake with improved FCR. Other studies, such as those replacing FM with microalgae in Atlantic salmon and European sea bass (*Dicentrarchus Labrax*) diets, have found similar results [48,49]. Recent studies have shown that taurine is crucial in improving growth rates, feed consumption, and feed utilization in fish, including trout [50,51,52,53]. Since diets containing FM are rich in taurine, exogenous taurine may be necessary in FM-free feed to maintain trout’s physiological functions and increase feed intake. Adding lecithin to aquafeed containing low levels of FM has also improved feed intake and growth rates in trout, salmon, and flounder (*Paralichthys olivaceus*) [54,55,56]. In this experiment, taurine and lecithin were used to enhance palatability and growth performance; and our results suggest these two additions may help replace FM with microalgal co-product meal in trout feed.

Even though nowadays the amount of FM used in diets for carnivorous species like rainbow trout has shown a clear decreasing trend toward a more selective use of FM as a strategic ingredient at lower levels, depending on the fish life-cycle stage and species of the fish, they contain highly variable amounts depending on the country and region. Plant-based ingredients constitute a significant portion of the fish feed used for various fish species, including rainbow trout. However, currently available sustainable fish feeds that are free of marine ingredients still need to be more efficient to ensure a reasonable economic income for fish farmers. Rainbow trout is a typically widely cultured carnivorous fish species and significant contributor to global aquaculture, with estimated production of 959,600 t in 2020 [1]. For example, most of the studies performed in fish nutrition replacing FM with microalgal biomass have been conducted using Reference (control) diets for rainbow trout with a wide ranges of FM use from 7.5 to 30% [37,42,57,58]. The reference diet formulated for this study contained low FM (7.5%) for rainbow trout. The remaining protein sources are combined with other sources, including feather meal (15%), blood meal (7%), corn gluten meal (20%), and soy protein concentrate (20%). In the previous studies we formulated an optimized diet, which showed excellent growth, feed, and filet quality [42,59]. Overall, the use of FM use in the aquafeed sector has continued to increase as a consequence of the growth in aquaculture production and the related consumption of aquafeeds. However, due to the current price of FM and increased pressure on the stressed marine resource, a further reduction in FM inclusion in aquafeeds is thus mandatory. This manuscript reports how varying level of inclusions of microalgal co product affected growth, FCR, PER and cost viability in rainbow trout. There needs to be more information in the literature regarding the growth, feed efficiency, and cost competitiveness of (*ac*) inclusions when feeding rainbow trout. Obtaining this data is crucial for creating a high-quality fishmeal-free diet with co-products for rainbow trout. Thus, it was compared (*ac*) inclusion diets to a reference diet with a composition already known to yield excellent growth and cost competitiveness.

It is well documented that methionine and lysine are the most limiting essential amino acids in current low fishmeal aquaculture diets with higher inclusion of terrestrial protein sources [29,60,61]. With the exception of phenylalanine and leucine, all other essential amino acids, including methionine, lysine, and arginine, were lower in the coproduct compared to FM (Table S1). Thus, the proper balance of protein and amino acids in each experimental diet required a higher amount of coproduct inclusions to replace FM (for example, 7.5% FM replaced with 10% coproduct), plus limited amino acids (methionine and lysine) were equally supplemented (including taurine) (Table 1) all the diet to ensure adequate levels of the essential amino acids that were most limiting in the protein blends, lysine, and methionine [41]. The proper balance of protein and amino acids in each experimental diet was corroborated and reflected by the equal amino acid profile of rainbow trout muscle (Table 3).

### 4.2. Effects of Microalgal Co-Product Feeds on Filet Proximate Composition, Fatty and Amino Acids Profiles

Salmonids can synthesize n-3- LC-PUFA but to a limited degree, therefore, they must get n-3-LC-PUFA from dietary sources [25,62]. Also, a diet containing elevated levels of dietary n-3-LC-PUFA could reduce energy expenditure that would have been spent on de novo DHA biosynthesis and consequently support fish growth performance [63]. Farmed salmonids are commonly promoted for their positive impact on human health, specifically because of their high levels of n-3 LC-PUFA. However, recently, EPA and DHA contents in filets of farmed salmonids have decreased due to feed manufacturers replacing fish ingredients with terrestrial plants in aquafeeds [64,65]. Terrestrial plants do not provide sufficient levels of these fatty acids to the fish and may contain harmful anti nutritional factors [66,67,68]. It is easier for salmonoids to consume and incorporate dietary long-chain fatty acids into their fillet fatty acid composition, rather than synthesizing short-chain fatty acids. In our testing of the (*ac*) microalgal co-product as a novel ingredient to replace FM ingredients in trout feeds, there was no significant differences detected in the total n-3-PUFA fatty acids, EPA, and DHA between the co-product experimental diets and the FM-based reference diet.

Although not significantly different, the similar content of n-3 PUFAs, especially the essential EPA & DHA deposition in trout fed the microalgal co-product diets, indicate that defatted (*ac*) biomass (still contains a small amount of EPA, 0.56% of TFA; Table S2) is a promising trout feed ingredient to replace FM. The beneficial effects of marine microalgae as a PUFA source in salmonids, including trout feed, have also been documented in other studies [7,69,70,71,72,73].

Several studies have shown that terrestrial crops also have deficient levels of rainbow trout essential amino acids such as lysine, methionine and tryptophan [28,29]. The essential amino acids in the rainbow trout filet fed the microalgae diet were not significantly different from the fish fed the reference diet and similar to previous studies that measured rainbow trout filet amino acid content [74,75]. Since amino acids are the building blocks of protein, rainbow trout diets need to offer complete essential amino acid composition to ensure that consumers are consuming high-quality protein.

### 4.3. Effects of Microalgae Replacement of FM on Filet Minerals

There were no significant differences between the macrominerals and trace elements in the filets of the fish fed the reference diet and (*ac*) co-product diets. Macronutrients and especially trace elements from filets are important to measure because their consumption can be health hazards to humans if they are above acceptable limits. Dietary ingredients can be a risk if they contain toxic trace elements that enter fish bodies and accumulate in tissue. Purchasing aquaculture feeds or feed ingredients from a country of origin with high dietary nutrient standards can prevent higher trace metal contamination. The trace elements detected in filets included iron, zinc, arsenic, mercury, and lead. FO is a known source of arsenic in commercial aquaculture feeds [42,76,77,78]. Little data highlights the effects of microalgae on the trace element composition of rainbow trout filets. The levels of the trace elements detected in filets (Table 6) were lower or at the same levels of trace elements reported from a previous study which replaced fish meal with insect meal in the diet of Atlantic salmon [79]. Also, the detected levels of trace elements in the filets were below the 0.10 mg kg^−1^ allowed in aquaculture feed set by the European Union [80]. Even though a low level of arsenic was detected in the 66N diet, this study shows that overall (*ac*) co-product diets pose no risk to the human consumption of heavy metals.

### 4.4. Effects of Microalgae on Economic Conversion Ratio

The 66N and 100N diets yielded lower ECRs, showing that (*ac*) can be a cost-competitive alternative feed ingredient to FM. The 33N diet had a lower feed cost but a higher FCR, resulting in an elevated ECR. According to a study by [81], the biofuels industry has the potential to lead research and development on microalgae culture in order to reduce feed expenses. One way to achieve this is by using waste streams and value-added co-products to decrease the cost of producing microalgal biomass. It is essential to increase research and development of cultivating and harvesting *Nannochloropsis*. Cultivation of marine microalgal species can use wastes from other industries, and does not compete for land that can be used for terrestrial food production [82,83,84,85,86]. Studies have also recently shown that aquaculture feeds can successfully incorporate microalgal co-products leftover from omega-3 fatty acid extraction to produce human nutritional supplements [36,42]. It has been recently reported that for Nile tilapia (*Oreochromis Niloticus*) feed, FM was completely replaced by *N. oculata* co-product, and FO was replaced by *Schizochytrium* oil; and estimated that microalgae-based feed costs less to produce a kg of tilapia than fish raised on conventional FM and FO-based feed [7].

In the current study, the cost of entirely replacing FM with microalgal co-product feed, 100N, was $0.88 per kg of diet for rainbow trout, which is less than the cost of the FM-based reference diet ($0.95 per kg of diet). The economic conversion ratio ($ per kg of trout production) is almost the same for trout fed the reference diet ($0.88 per kg of trout) and trout fed fully replaced FM using microalgal co-product feed ($0.86 per kg of trout) due to slightly higher FCR in the microalgal co-product feed compared to the reference feed. Based on various estimates, it has been found that using microalgae as feed is lower cost than using FM (which costs $1.5 per kg) or insect-based feed (which costs between $3 to $5.9 per kg). However, for microalgal co-product feed tested in this study, 100N is more expensive than plant-based feed, (which costs $0.64 per kg). These estimates have been published by [42,87,88]. It is predicted that with the emergence of large-scale facilities, the cost of microalgal biomass and feed will decrease, making it more competitive [88]. In addition, when conducting economic analyses, it is important to take into account the expenses associated with eutrophication and other emissions that stem from the entire lifecycle of each ingredient, starting from production all the way to excretion. Some recent studies [46,82,89] have explored the development of life cycle analyses (LCA) for *Nannochloropsis*. It is crucial now to better document the scalability of these analyses towards larger production and to conduct scenario assessments.

One of the limitations of this study in estimating ECR is that the costs of taurine and lecithin were not considered in the economical evaluation. It should be noted that taurine and lecithin were included across the diets including reference and all FM replacing microalgal co-product diet, however it did not reflect the estimated diet costs. Although ECR value showed promising this study, one of the biggest challenges is competitive price of microalgal ingredients with conventional ingredients. Price is the major constraint, bulk supply from scale-up that will dictate the future course of the aquafeed sector and industry [11]. It is important to note that the cost of producing microalgal meals is much higher than fishmeal. For example, the production cost of autotrophically grown microalga (*Spirulina* and *Chlorella*) meal ranges from approximately 10 USD/kg to 30 USD/kg [90]. On the other hand, the highest price achieved for fishmeal in 2013 was just 1.74 USD/kg. Therefore, due to the significant price difference, it is currently unrealistic for microalgal meals to replace fishmeal. Currently, the world’s production of autotrophic microalgae biomass is approximately 20,000 tons (dry weight), according to Benemann et al. (2018) [90]. On the other hand, the annual production of fishmeal used in aquaculture is estimated to be 3,900,000 tons [91]. As a result, the production gap between the two is quite significant and needs to be bridged. Major challenge is to generating consistency in the supply of the microalgal ingredients to produce large quantities on an industrial scale. Thus, scaling up is a real block for microalgal ingredients. Moreover, microalgal production costs are much higher than fishmeal. However, aquaculture feed manufacturers are willing to pay a similar price per ton as soy protein concentrate as opposed to fishmeal. As traditional fish feed ingredients make up a significantly large portion of the aquafeed today, the focus has just begun to include minor percentage of new raw ingredients because still there is no any solid guarantee for consistent supplies and with competitive costs. Researchers and microalgae R&D have to find the ways to lower costs of producing and processing of novel ingredients. For example, although replacing fish oil with microalgae showed promise, producing microalgae and extracting oil requires access to advanced technologies, expertise, and costs. Despite above limitations, the stakeholders agreed to adopt novel ingredients for aquafeeds where it was feasible would be an important step towards reducing pressure on wild-caught fish in the aquaculture diet that could secure the future sustainability of the aquaculture sector.

Expanding the microalgal raw material portfolio through innovation will allow flexibility in formulations with focus on optimal nutrition, availability, sustainability, and cost [88]. To achieve this potential there should be a focus on improving the scale of production, which will ensure the process chain is environmentally sustainable and reduce the cost of production. The biological capacity of microalgae, underpinned with positive research findings on the replacement efficacy in aquafeeds across many aquaculture species, suggests that there is high potential for the use of microalgae as a protein source. However, scaling up the continuous production of high-quality microalgal biomass and its downstream processing requires addressing technical, biological, and economic challenges [92,93]. In recent years, there has been a rise in the construction of large-scale cultivation facilities. Our industry collaborator, Qualitas Health Inc., a US leader in commercial microalgae nutraceuticals, generates tons of *Nannochloropsis* co-product at its farms in Columbus, New Mexico (364 ha, 98 of which currently cultivated) and Imperial, Texas (145 ha, 45 of which currently cultivated). The company plans to expand the Columbus farm and Imperial farm in the next few years. According to various estimates, these large facilities can produce microalgal biomass at even lower costs. For example, a 405 ha open raceway pond study found that microalgae biomass production can be much lower with large volumes [92]. As large-scale microalgae-based industries continue to emerge, the cost of microalgae-based feed is expected to decrease further, making it cost-competitive aquaculture feed. Therefore, it is essential to develop methods and strategies for larger-scale and lower-cost production of various resources using open-field systems, as this could be viable option.

### 4.5. Effects of Microalgae on Apparent Digestibility

The defatted (*ac*) showed improved macronutrient, amino acid, and fatty acid fraction apparent digestibility above 90% ADC (Table 8). Most of the apparent digestibility values of the macronutrients, amino acids, and fatty acid fractions were higher than previously reported ranges for whole cells of (*ac*) in rainbow trout [7], *Nannochloropsis* sp. replacement in Atlantic salmon [34,35,58] and *Nannochloropsis* sp. in European sea bass [94]. It is unknown to obtain mean values of lipid digestibility of 99% when values of SFA and MUFA were much below. However, higher digestibility values of long-chain n-3 and n-6 PUFA, EPA, and DHA can partly explain higher crude lipid digestibility. Previous studies have found ADCs of macronutrient, amino acid, and fatty acid fractions of alternative ingredients, including microalgae and vital wheat gluten, to be in the 99% range [95,96]. The apparent digestibility of lipid was reported 92.5% in rainbow trout [97]. It is surprising to obtain a value of 99% digestibility for all major nutrients, even crude fiber, or more than 80% for minerals. This may be due to the extensive sieving of the plant meals and microalgal co-product and then extrusion processing of the diets that occurred during the formulation of the experimental diets, which increases the digestibility of the whole diet by reducing indigestible fiber content. Also, the significant increase in diet ADC indicates that the fish gut microflora may have adapted to this dietary stressor by increasing in population in the presence of the elevated level of dietary fiber. However, there is no evidence in the literature to support this claim. The differences in ADC values of feed do occur frequently. They are usually the result of species differences, variations in the season of harvest/catch of the raw materials, and processing conditions used by various production plants. It was beyond control over these factors in the present study.

These higher digestibility results in our current study compared to reported values in the above studies could also be attributed to the following three main factors: differences in feed manufacturing technique (cold pellet vs. extruded pellet), fecal collection techniques, water temperatures, the type of microalgal ingredients (whole cells vs. lipid extracted co-product) used in the diet, and the microalgal ingredients inclusion level in the diet.

In this study, the feed was prepared using a thermal extruder that involves high temperature, high pressure, and processing time that could destroy the anti-nutrients (non-starch polysaccharides) and increase nutrient digestibility [11,34,35,98,99]. Previously it has been reported the digestibility of whole cells *Nannochloropsis* sp. in rainbow trout for a cold pelleted diet, unlike extruded pellet, which did not use high temperatures or pressure; thus the anti-nutrient composition in feed ingredients, including rigid cell wall *Nannochloropsis* sp. with complex carbohydrates (e.g., non-starch polysaccharides/fibers including cellulose, gums, pectins, and hemicelluloses) was largely unaffected during the manufacturing process [7] and displayed lower digestibility than in the current study. The higher digestibility value reported in our current study compared to Atlantic salmon and Seabass studies mentioned above could be mainly due to lower inclusion levels of *Nanochloroposis* co-product in our current study; that is the higher level of algal inclusion in the Atlantic salmon and Seabass might cause lower digestibility.

It is generally assumed that temperature affects digestibility, and several studies have demonstrated that digestibility increases with rising water temperature [100,101]. Higher temperature has been shown to increase metabolism, leading to elevated enzyme activity, greater lipase production in the fish midgut, and faster absorption [102,103,104]. Our study results indicate that the digestibility at a water temperature of 15.4 °C may be attributed to the increased basal metabolic rate at this temperature [105]. For instance, Atlantic salmon kept at very low temperature (3 °C) exhibited significantly reduced digestibility [106].

**Figure 1:**
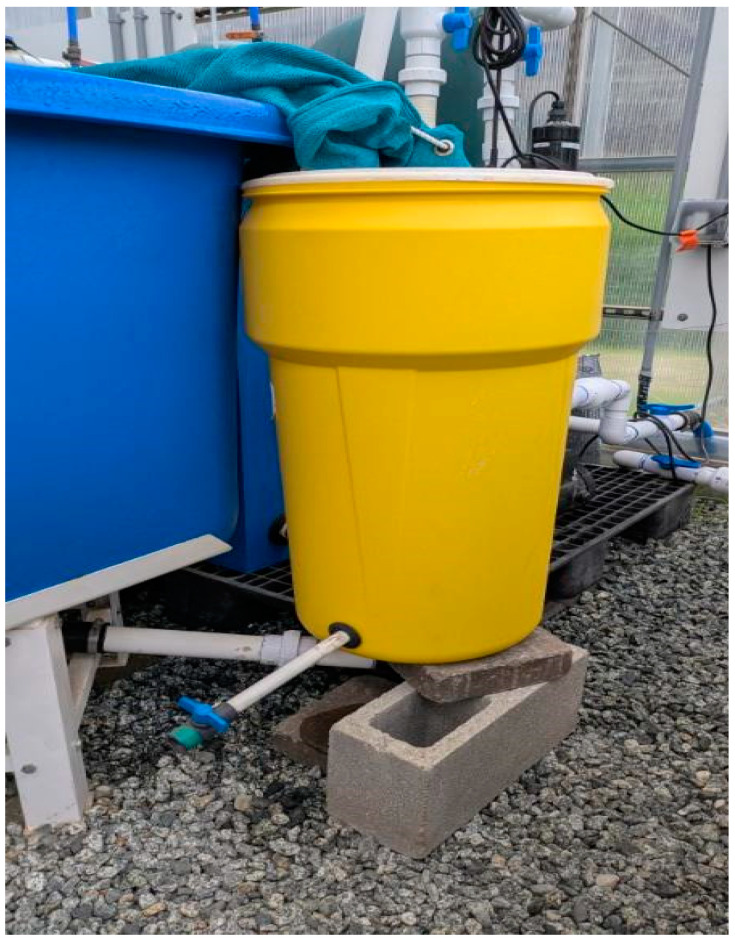
Radial flow settler (yellow bucket) attached to culture tank (Front blue bin).

It is widely recognized that different fecal collection techniques affect digestibility estimates, making comparisons between experiments difficult [107,108,109]. In addition, differences can occur due to procedures used by various laboratories, including the fecal collection method and variations in the formulation of the reference diet. The accuracy of ADC measurement in fecal samples can be affected by the method used for collection. Some methods can result in an underestimation of ADC, while others can lead to an overestimation due to leaching losses. It is well-documented that certain methods of collecting fecal samples, such as manual stripping, anal suction, or dissection, can cause significant stress to the animal. These methods may result in fecal samples contaminated with non-fecal nutrients, such as digestive enzymes, bodily fluids, sloughed epithelial cells, and other substances. This contamination can artificially increase the nutrient content of the sample, resulting in an underestimation of digestibility [110]. It has been opted in this study to use the settlement column technique utilized in the original Guelph system, as Cho et al. (1982) reported [45], which has shown no significant leaching losses. In addition, our modified radial flow settler design further reduced the likelihood of leaching losses by increasing fecal recovery time and collecting intact fecal matter at the bottom of the system by installing a radial flow settler between the culture tank outflow and the sump tank inflow [12] (Figure 1). In earlier work, it was confirmed the use of method for our work with rainbow trout [12,42]. Thus, although discrepancies exist among methods, and even assuming some overestimation of digestibility, the settlement column is still considered one of the preferred methods for determining digestibility in fish.

## 5. Conclusions

The overall performance of rainbow trout juveniles showed that incorporating (*ac*) ingredients may be a practical approach to fully replace FM in rainbow trout feed. Specifically, the (*ac*) tested in this study can entirely eliminate FM in trout feeds without compromising key performance indicators. This approach to feed formulation in entirely replacing FM with microalgal co-product can be sustainable for trout aquaculture in that it was found equal fish growth; maintenance of protein, amino acids, macro, and micro minerals, and n-3 PUFAs content in the fish filet; and cost-competitiveness via a lower economic conversion ratio (in the 66N and 100N diets) compared with an FM based conventional trout feed. Although microalgal species have been used for replacing either FM or FO, their full potential to combine different species of microalgae has not yet been realized. Towards this end, our ongoing research focuses on developing microalgae-based fish-free feed for sustainable rainbow trout aquaculture. Further research to also determine the optimum inclusion level of (*ac*) for adult rainbow trout is necessary to evaluate suitability of microalgal co-product diet to all stages of the production cycle of the species.

## Figures and Tables

**Table 1:** Formulation (g/100 g diet) and essential amino acids (% in the weight of diet) of four experimental diets for rainbow trout.

	Diet			
Ingredient (%)	Reference ^1^	33N ^2^	66N ^3^	100N ^4^
FM ^5^	7.50	5.02	2.55	0.00
Fish oil	14.00	14.00	14.00	14.00
Co-product meal (raw)	0.00	4.10	7.40	10.00
Feather meal	15.00	15.00	15.00	15.00
Blood meal	7.00	7.00	7.00	7.00
Corn gluten meal	20.00	20.00	20.00	20.00
Soy protein concentrate	20.00	20.00	20.00	20.00
Wheat gluten	5.00	5.00	5.00	5.00
CaHPO_4_	1.00	1.00	1.00	1.00
Vitamin-mineral premix ^6,7^	0.60	0.60	0.60	0.60
Lysine	1.00	1.00	1.00	1.00
Methionine	0.20	0.20	0.20	0.20
Choline chloride	0.50	0.50	0.50	0.50
Alpha cellulose	4.50	2.89	2.00	2.00
Ascorbic acid	0.20	0.20	0.20	0.20
Taurine	0.50	0.50	0.50	0.50
Lecithin	3.00	3.00	3.00	3.00
Astaxanthin	0.05	0.05	0.05	0.05
Proximate composition (%)				
Moisture	17.08 ± 0.71	18.49 ± 0.01	13.21 ± 0.14	12.86 ± 0.12
Protein	48.16 ± 0.24	47.70 ± 0.17	49.39 ± 0.15	49.18 ± 0.05
Fat	15.89 ± 0.08	15.66 ± 0.04	18.01 ± 0.17	17.15 ± 0.08
Fiber	3.53 ± 0.45	2.47 ± 0.21	2.18 ± 0.04	2.39 ± 0.03
Ash	5.57 ± 0.17	5.77 ± 0.07	6.30 ± 0.01	6.63 ± 0.01
Carbohydrates	13.29 ± 0.76	12.37 ± 0.29	13.09 ± 0.29	14.18 ± 0.08
Energy (kJ g^−1^)	3378.33 ± 24.26	3347.67 ± 7.88	3640.67 ± 10.81	3591.67 ± 8.01
Amino acids (% in the weight of diet as is)				
Arginine	2.73 ± 0.06	2.74 ± 0.03	2.84 ± 0.06	2.85 ± 0.02
Histidine	1.05 ± 0.01	1.08 ± 0.02	1.13 ± 0.02	1.17 ± 0.04
Isoleucine	1.74 ± 0.12	1.82 ± 0.09	1.82 ± 0.02	1.89 ± 0.06
Leucine	4.48 ± 0.06	4.59 ± 0.04	4.73 ± 0.02	4.89 ± 0.03
Lysine	2.78 ± 0.04	2.93 ± 0.16	3.08 ± 0.07	3.12 ± 0.06
Methionine	0.75 ± 0.02	0.78 ± 0.02	0.84 ± 0.01	0.82 ± 0.00
Phenylalanine	2.50 ± 0.03	2.61 ± 0.03	2.69 ± 0.01	2.75 ± 0.02
Threonine	1.92 ± 0.00	1.83 ± 0.01	1.86 ± 0.03	1.91 ± 0.03
Valine	2.46 ± 0.14	2.61 ± 0.12	2.55 ± 0.00	2.66 ± 0.11

^1^ Reference: no replacement of FM (FM) and fish oil (FO). ^2^ Replacement of 33% of FM with with (*ac*). ^3^ Replacement of 66% of FM with with (*ac*). ^4^ Replacement of 100% of FM with with (*ac*). ^5^ Omega Protein, Inc., Houston, TX 77042, USA, as manufacturer specification, the guaranteed gross composition analysis: crude protein, 60%; crude fat, 6%; fiber, 2%. ^6^ Mineral premix (mg kg^−1^ dry diet unless otherwise stated): ferrous sulphate, 0.13; NaCl, 6.15; copper sulphate, 0.06; manganese sulphate, 0.18; potassium iodide, 0.02; zinc sulphate, 0.3; carrier (wheat middling or starch). ^7^ kVitamin premix (mg kg^−1^ dry diet unless otherwise stated):vitamin A (as acetate), 7500 IU kg^−1^ dry diet; vitamin D3 (as cholecalcipherol), 6000 IU kg^−1^ dry diet; vitamin E (as DL-a-tocopherylacetate), 150 IU kg^−1^ dry diet; vitamin K (as menadione Na-bisulphate), 3; vitamin B12 (as cyanocobalamin), 0.06; ascorbic acid (as ascorbyl polyphosphate), 150; D-biotin, 42; choline (as chloride), 3000; folic acid, 3; niacin (as nicotinic acid), 30; pantothenic acid, 60; pyridoxine, 15; riboflavin, 18; thiamin, 3.

**Table 2:** Growth indices (mean ± standard error, *n* = 4) of rainbow trout fed experimental diets for 64 days.

	Filet ^1^				ANOVA ^2^	
	Reference	33N	66N	100N	*F* Value	*p* Value
Initial Wt. (g)	40.90 ± 0.83	39.70 ± 0.39	40.00 ± 0.43	41.20 ± 1.02	1.02	0.41
Final Wt. (g)	117.00 ± 3.30	111.00 ± 1.42	113.00 ± 1.81	114.00 ± 1.96	1.38	0.29
Wt. gain (g) ^3^	76.20 ± 2.84	71.0 ± 1.26	73.3 ± 1.72	72.80 ± 1.08	1.33	0.30
Wt. gain (%) ^4^	186.00 ± 6.26	179.00 ± 3.16	183.00 ± 4.61	177.00 ± 3.00	0.90	0.46
FCR ^5^	0.93 ± 0.04	1.01 ± 0.01	0.97 ± 0.02	0.98 ± 0.02	1.49	0.26
SGR ^6^	0.95 ± 0.05	0.90 ± 0.03	0.93 ± 0.04	0.88 ± 0.03	0.86	0.48
PER ^7^	2.24 ± 0.08	2.08 ± 0.03	2.08 ± 0.04	2.08 ± 0.05	1.98	0.17
Feed intake (g/fish)	70.80 ± 0.04	71.58 ± 0.61	71.20 ± 0.53	71.08 ± 0.65	0.39	0.76
Survival (%) ^8^	100.00	100.00	100.00	100.00		

^1^ Mean ± Standard Error (n = 4 replicates per diet; pooled whole tissues of 5 fish/replicate). ^2^ ANOVA test showed no significant differences between treatments in all metrics (*p* > 0.05). ^3^ Weight (Wt.) gain (g) = final Wt. − initial Wt. ^4^ Wt. gain (%) = (final Wt. − initial Wt.)/initial Wt. × 100. ^5^ Feed conversion ratio (FCR) = feed intake (g)/Wt. gain (g). ^6^ Specific growth rate (SGR) (%/day) = 100% × (ln final wet Wt. (g) − ln initial wet Wt. (g))/Time (days). ^7^ Protein efficiency ratio (PER) = Wt. gain (g)/protein fed (g). ^8^ Survival (%) = (Final number of fish/Initial number of fish) × 100%.

**Table 3:** Essential amino acid content (dry weight basis) of filets from rainbow trout after 64 days on the experimental diets.

Essential Amino Acids (%)	Filet ^1^				ANOVA	
	Reference	33N	66N	100N	*F* Value	*p* Value
Methionine	2.14 ± 0.09	2.06 ± 0.15	2.11 ± 0.09	2.01 ± 0.08	0.32	0.80
Lysine	6.68 ± 0.28	6.49 ± 0.30	6.47 ± 0.33	6.39 ± 0.18	0.19	0.89
Phenylalanine	2.78 ± 0.09	2.68 ± 0.11	2.67 ± 0.12	2.62 ± 0.09	0.39	0.75
Leucine	5.43 ± 0.22	5.28 ± 0.20	5.26 ± 0.24	5.19 ± 0.17	0.21	0.88
Isoleucine	2.78 ± 0.28	2.63 ± 0.19	2.55 ± 0.29	2.42 ± 0.17	0.41	0.74
Threonine	3.36 ± 0.14	3.21 ± 0.13	3.18 ± 0.15	3.17 ± 0.15	0.36	0.77
Valine	3.11 ± 0.29	2.94 ± 0.19	2.86 ± 0.26	2.66 ± 0.19	0.60	0.62
Histidine	1.96 ± 0.11	1.96 ± 0.07	1.92 ± 0.09	1.93 ± 0.08	0.05	0.98
Arginine	4.48 ± 0.12	4.40 ± 0.12	4.32 ± 0.21	4.20 ± 0.10	0.70	0.56
Tryptophan	0.61 ± 0.06	0.47 ± 0.08	0.57 ± 0.06	0.57 ± 0.07	0.82	0.50

^1^ Mean ± Standard Error (n = 4 replicates per diet; pooled whole tissues of 5 fish/replicate).

**Table 4:** Amounts of lipid and major omega-3 (n-4) and n-6 polyunsaturated fatty acid (PUFA) in the trout filet (wet weight basis) of rainbow trout-fed experimental diets for 64 days.

Filet PUFA (mg FA g^−1^)	Filet PUFA ^1^				ANOVA	
	Reference	33N	66N	100N	*F* Value	*p* Value
18:2n6 LA ^2^	21.30 ± 1.78	23.9 ± 0.81	21.6 ± 1.80	22.60 ± 2.64	0.40	0.75
20:4n6 ARA ^3^	1.32 ± 0.20	1.42 ± 0.04	1.32 ± 0.18	1.30 ± 0.25	0.09	0.97
18:3n-3 ALA ^4^	2.34 ± 0.23	2.98 ± 0.11	2.50 ± 0.29	2.68 ± 0.48	0.78	0.53
20:5n-3 EPA ^5^	7.69 ± 1.80	8.40 ± 0.73	6.85 ± 1.08	6.60 ± 1.29	0.41	0.75
22:6n-3 DHA ^6^	14.90 ± 3.29	18.40 ± 0.98	14.00 ± 2.20	14.70 ± 3.44	0.57	0.65

^1^ Mean ± Standard Error (n = 4 replicates per diet; pooled whole tissues of 5 fish/replicate). ^2^ Linoleic Acid (LA). ^3^ Arachidonic acid (ARA). ^4^ Alpha linolenic acid (ALA). ^5^ Eicosapentaenoic acid (EPA). ^6^ Docosahexaenoic acid (DHA).

**Table 5:** The fatty acid content of filets from rainbow trout after 64 days on the experimental diets.

	Filet ^1^				ANOVA	
Filet (mg FA/g)	Reference	33N	66N	100N	*F* Value	*p* Value
14:00	6.93 ± 0.78	6.24 ± 0.33	7.05 ± 0.62	6.99 ± 0.74	0.34	0.80
15:00	0.69 ± 0.07	0.62 ± 0.03	0.70 ± 0.05	0.70 ± 0.06	0.47	0.71
16:00	31.27 ± 2.72	30.95 ± 1.58	32.10 ± 2.05	32.20 ± 2.54	0.07	0.97
17:00	0.79 ± 0.08	0.69 ± 0.03	0.84 ± 0.05	0.84 ± 0.08	1.14	0.37
18:00	7.23 ± 0.62	7.15 ± 0.3	7.40 ± 0.35	7.33 ± 0.60	0.05	0.98
20:00	0.38 ± 0.03	0.43 ± 0.02	0.41 ± 0.03	0.42 ± 0.03	0.66	0.59
22:00	0.20 ± 0.03	0.21 ± 0.00	0.23 ± 0.01	0.23 ± 0.02	0.76	0.54
24:00:00	0.19 ± 0.01	0.2 ± 0.01	0.21 ± 0.01	0.22 ± 0.02	0.94	0.45
Total SFA ^2^	47.71 ± 4.32	46.57 ± 2.2	48.98 ± 3.14	48.97 ± 4.05	0.11	0.95
16:1n-9	0.41 ± 0.04	0.43 ± 0.03	0.40 ± 0.03	0.39 ± 0.03	0.37	0.78
16:1n-7	11.88 ± 1.54	10.68 ± 0.52	12.23 ± 1.07	11.84 ± 1.21	0.35	0.79
18:1n-9	24.55 ± 1.56	32.46 ± 2.27	25.71 ± 3.19	27.01 ± 3.45	1.65	0.23
18:1n-7	4.86 ± 0.44	5.25 ± 0.27	4.96 ± 0.32	4.90 ± 0.41	0.23	0.87
20:1n-9	1.45 ± 0.12	1.91 ± 0.23	1.39 ± 0.15	1.39 ± 0.16	2.22	0.14
20:1n-7	0.21 ± 0.03	0.22 ± 0.03	0.20 ± 0.02	0.17 ± 0.02	0.64	0.61
22:1n-11	0.31 ± 0.07	0.31 ± 0.04	0.30 ± 0.05	0.25 ± 0.06	0.29	0.83
22:1n-9	0.38 ± 0.06	0.41 ± 0.02	0.36 ± 0.03	0.33 ± 0.03	0.75	0.54
24:1n-9	45.10 ± 2.59	54.28 ± 3.95	45.98 ± 3.98	46.71 ± 4.22	1.28	0.33
Total MUFA ^3^	21.25 ± 1.78	23.90 ± 0.81	21.61 ± 1.80	22.62 ± 2.64	0.40	0.75
18:2n-6	0.38 ± 0.04	0.48 ± 0.04	0.41 ± 0.07	0.4 ± 0.07	0.70	0.57
18:3n-6	0.88 ± 0.05	1.03 ± 0.04	0.91 ± 0.05	0.96 ± 0.14	0.65	0.60
20:2n-6	0.54 ± 0.06	0.65 ± 0.03	0.55 ± 0.07	0.56 ± 0.1	0.54	0.66
20:3n-6	1.32 ± 0.20	1.42 ± 0.04	1.32 ± 0.18	1.30 ± 0.25	0.09	0.97
20:4n-6 ARA ^4^	0.18 ± 0.04	0.17 ± 0.01	0.15 ± 0.02	0.16 ± 0.02	0.19	0.90
22:4n-6	0.59 ± 0.09	1.01 ± 0.07	0.69 ± 0.14	0.87 ± 0.25	1.45	0.28
22:5n-6	25.13 ± 2.10	28.67 ± 0.96	25.65 ± 2.28	26.87 ± 3.45	0.44	0.73
Total n-6 PUFA ^5^	2.34 ± 0.23	2.98 ± 0.11	2.50 ± 0.29	2.68 ± 0.48	0.78	0.53
18:3n-3 ALA ^6^	1.44 ± 0.28	1.60 ± 0.17	1.32 ± 0.19	1.27 ± 0.23	0.45	0.72
18:4n-3	0.22 ± 0.04	0.26 ± 0.01	0.22 ± 0.03	0.23 ± 0.05	0.37	0.78
20:3n-3	1.13 ± 0.26	1.25 ± 0.06	1.01 ± 0.13	1.10 ± 0.23	0.30	0.83
20:4n-3	7.69 ± 1.80	8.40 ± 0.73	6.85 ± 1.08	6.60 ± 1.29	0.41	0.75
20:5n-3 EPA ^7^	2.57 ± 0.66	2.81 ± 0.2	2.41 ± 0.38	2.29 ± 0.49	0.24	0.87
22:5n-3	14.87 ± 3.29	18.43 ± 0.98	13.97 ± 2.20	14.65 ± 3.44	0.57	0.65
22:6n-3 DHA ^8^	30.72 ± 6.60	36.24 ± 2.19	28.68 ± 4.26	29.24 ± 6.26	0.45	0.72
Total n-3 PUFA ^9^	58.28 ± 8.67	67.12 ± 2.75	56.69 ± 6.37	58.43 ± 9.98	0.40	0.76
Total PUFA	3.50 ± 0.35	4.29 ± 0.13	3.63 ± 0.44	3.85 ± 0.75	0.52	0.68
Total n-6 LCPUFA ^10^	26.94 ± 6.13	31.66 ± 1.96	24.86 ± 3.82	25.29 ± 5.54	0.45	0.72
Total n-3 LCPUFA ^11^	1.20 ± 0.22	1.27 ± 0.07	1.11 ± 0.12	1.04 ± 0.12	0.45	0.73
n-3/n-6 PUFA ratio ^12^	7.39 ± 1.29	7.39 ± 0.43	6.82 ± 0.68	6.43 ± 0.27	0.37	0.78

^1^ Total fatty acids (TFA) (%); Mean ± standard error for 4 replicates per diet (pooled whole tissues of 5 fish/replicate). ^2^ Saturated fatty acids (SFA) is the sum of all fatty acids without double bonds. ^3^ Monounsaturated fatty acids (MUFA) is the sum of all fatty acids with a single bond. ^4^ Arachidonic acid (ARA). ^5^ Omega-6 (n-6) Polyunsaturated fatty acids (PUFAs) (sum of all fatty acids with ≥2 double bonds (18:2, 18:3, 20:2, 20:3, 20:4, 22:4, 22:5). ^6^ Alpha-linolenic acid (ALA). ^7^ Eicosapentaenoic acid (EPA). ^8^ Docosahexaenoic acid (DHA). ^9^ Omega-3 (n-3) PUFAs (18:3, 18:4, 20:3, 20:4, 20:5, 22:5, 22:6). ^10^ n-6 long-chain (LC) PUFA (20:2, 20:3, 20:4, 22:4, 22:5). ^11^ n-3 LCPUFA (20:3, 20:4, 20:5, 22:5, 22:6). ^12^ Ratio calculated for total n-3 PUFA: total n-6 PUFA (n-3/n-6).

**Table 6:** Macro minerals (%) and trace elements content (mg/kg) (wet weight basis) of filet from rainbow trout after 64 days on the experimental diets.

Macro Minerals (%)	Filet ^1^				ANOVA ^2^	
	Reference	33N	66N	100N	*F* Value	*p* Value
Phosphorus	2.17 ± 0.03	2.26 ± 0.01	2.14 ± 0.04	2.12 ± 0.06	2.16	0.14
Calcium	0.34 ± 0.06	0.38 ± 0.04	0.22 ± 0.01	0.28 ± 0.03	3.02	0.07
Magnesium	0.22 ± 0.00	0.23 ± 0.00	0.22 ± 0.01	0.21 ± 0.01	1.13	0.37
Potassium	3.45 ± 0.09	3.57 ± 0.07	3.49 ± 0.11	3.37 ± 0.09	0.92	0.45
Sulfur	1.60 ± 0.02	1.63 ± 0.03	1.64 ± 0.03	1.61 ± 0.02	0.48	0.69
Trace elements (mg kg^−1^)						
Copper	ND	ND	ND	ND		
Iron	0.07 ± 0.00	0.08 ± 0.00	0.07 ± 0.00	0.07 ± 0.00	1.80	0.19
Manganese	ND	ND	ND	ND		
Selenium	ND	ND	ND	ND		
Zinc	0.02 ± 0.00	0.02 ± 0.00	0.02 ± 0.00	0.01 ± 0.00	2.74	0.08
Arsenic	0.001 ± 0.001	0.001 ± 0.001	0.002 ± 0.001	0.001 ± 0.001	2.58	0.10
Boron	ND	ND	ND	ND		
Aluminum	ND	ND	ND	ND		
Mercury	0.01 ± 0.00	0.01 ± 0.00	0.01 ± 0.00	0.01 ± 0.00	0.15	0.92
Lead	0.009 ± 0.00 ^ab^	0.010 ± 0.00 ^ab^	0.012 ± 0.00 ^a^	0.008 ± 0.00 ^b^	3.99	0.03
Molybdenum	ND	ND	ND	ND		

^1^ Values are means ± standard errors of four replicate groups (n = 4); each replicate involving pooled whole tissues of 5 fish. ^2^ Mean values not sharing a superscript letter in the same row differ significantly (*p* < 0.05) from Tukey’s HSD test. Not detectable (ND) (<0.000 μg/g).

**Table 7:** Formulated feed cost, feed conversion ratio, and economic conversion ratio of rainbow trout production ^1^.

Scenario	Formulated Feed Cost	Feed Conversion Ratio ^2^	Economic Conversion Ratio ^3^
	(US$/kg feed)		($/kg rainbow trout)
Reference	0.95	0.93	0.88
33N	0.93	1.01	0.94
66N	0.91	0.97	0.88
100N	0.88	0.98	0.86
*F* value ^5^	NA ^4^	1.49	2.11
*p* value	NA ^4^	0.26	0.14

^1^ Mean ± standard error for four replicates per diet. ^2^ Feed conversion ratio (FCR) = feed intake (g)/Wt. gain (g). ^3^ ECR ($/kg fish) = FCR ((kg diet fed)/(kg weight gain)) × price of diet (USD$/kg diet). ^4^ Not applicable ANOVA testing was not needed. ^5^ ANOVA test showed no significant differences between treatments in all metrics (*p* > 0.05).

**Table 8:** Apparent digestibility coefficients (%, mean ± standard error, n = 4) of nutrients in the reference diet and test diets for rainbow trout.

Nutrient	Diet Ingredients ^1^				ANOVA ^2^	
	Reference	33N	66N	100N		
Proximate Composition					F-Value	*p*-Value
Dry matter	99.25 ± 0.05 ^a^	99.52 ± 18.4 ^b^	99.09 ± 13.09 ^a^	99.14 ± 12.75 ^a^	18.842	<0.001
Crude protein	99.28 ± 0.03	99.29 ± 47.36	99.2 ± 48.99	99.26 ± 48.82	1.096	0.395
Lipid	98.89 ± 0.08	99.13 ± 15.52	98.98 ± 17.82	99 ± 16.98	1.234	0.348
Ash	86.6 ± 0.41	86.23 ± 4.98	83.58 ± 5.27	84.65 ± 5.62	3.263	0.068
Crude fiber	99.13 ± 0.04 ^a^	99.39 ± 15.56 ^b^	99.4 ± 17.9 ^b^	99.43 ± 17.05 ^b^	9.036	0.003
Energy	97.97 ± 0.09 ^a^	98.67 ± 0.03 ^b^	98.45 ± 0.11 ^b^	99.98 ± 0 ^c^	130.477	<0.001
Essential amino acids						
Arginine	99.28 ± 0.08	99.44 ± 0.03	99.43 ± 0.02	99.48 ± 0.04	2.93	0.086
Lysine	99.36 ± 0.04	99.46 ± 0.02	99.42 ± 0.03	99.47 ± 0.04	2.416	0.127
Isoleucine	99.28 ± 0.04	99.33 ± 0.02	99.24 ± 0.05	99.31 ± 0.06	0.693	0.577
Leucine	99.5 ± 0.05	99.51 ± 0.02	99.46 ± 0.03	99.54 ± 0.03	0.834	0.505
Histidine	99.42 ± 0.05	99.51 ± 0.01	99.49 ± 0.02	99.54 ± 0.04	1.917	0.191
Methionine	99.22 ± 0.05	99.26 ± 0.03	99.21 ± 0.1	99.24 ± 0.07	0.126	0.942
Phenylalanine	98.21 ± 0.16	98.3 ± 0.06	98.19 ± 0.1	98.4 ± 0.11	0.637	0.608
Threonine	98.32 ± 0.08	98.28 ± 0.04	98.16 ± 0.09	98.33 ± 0.11	0.946	0.455
Tryptophan	99.57 ± 0.03	99.45 ± 0.07	99.46 ± 0.05	99.5 ± 0.06	1.335	0.317
Valine	97.68 ± 0.21	98.1 ± 0.1	97.91 ± 0.13	98.2 ± 0.14	2.123	0.161
Fatty acid fractions ^3^						
Total SFA	29.11 ± 9.08 ^a^	73.59 ± 1.89 ^b^	70.14 ± 5.55 ^b^	66.23 ± 6.16b	11.431	0.001
Total MUFA	60.27 ± 5.08	52.88 ± 3.53	37.9 ± 11.61	44.38 ± 9.92	1.774	0.215
Total PUFA	79.73 ± 2.89 ^a^	90 ± 1.08 ^b^	90.53 ± 1.38 ^b^	90.12 ± 1.5b	7.523	0.006
20:5n3 EPA	96.64 ± 0.5	98.27 ± 0.2	97.68 ± 0.39	97.27 ± 0.58	2.929	0.86
22:6n3 DHA	95.64 ± 0.68 ^a^	98.04 ± 0.21 ^b^	97.71 ± 0.34 ^b^	97.64 ± 0.3 ^ab^	6.332	0.011
Total n3 PUFA	89.92 ± 1.52 ^a^	95.53 ± 0.48 ^b^	95.53 ± 0.61 ^b^	95.25 ± 0.72 ^b^	8.123	0.005
Total n6 PUFA	90.82 ± 1.17 ^a^	94.65 ± 0.6 ^b^	95.24 ± 0.7 ^b^	95.15 ± 0.72 ^b^	6.246	0.012

^1^ Mean ± standard error for four replicates per diet. Letters appearing together means no difference. ^2^ Mean values not sharing a superscript letter in the same row differ significantly (*p* < 0.05) from Tukey’s HSD test. ^3^ SFA refers to saturated fatty acids; MUFA, monounsaturated fatty acids; PUFA, polyunsaturated fatty acids; EPA, eicosapentaenoic acid; DHA, docosahexaenoic acid.

## Data Availability

The original contributions presented in the study are included in the article/Supplementary Materials, further inquiries can be directed to the corresponding author.

## Supplementary Materials

The following supporting information can be downloaded at: https://www.mdpi.com/article/10.3390/foods14050781/s1, Table S1: Proximate composition and essential amino acids of *Nannochloropsis* sp. QH25 and fishmeal; Table S2: Fatty acid content (% of total fatty acids) defatted *Nannochloropsis* sp. QH25 co-product biomass used in the experimental diets; Table S3: Macrominerals and trace elements in the defatted *Nannochloropsis* sp. QH25 co-product biomass; Table S4: Fatty acid (% of total fatty acids) content of the experimental diets; Table S5: Macrominerals and trace elements in the experimental diets; Table S6: Experimental ingredient market prices obtained from Cruz Aquafeed Sustainability Tool (CAST); Table S7: Whole body proximate composition (wet weight basis) of rainbow trout after 64 days on the experimental diets; Table S8: Proximate filet composition (wet weight basis) of rainbow trout fed experimental diets for 64 days.
