# Supplementary material for: Towards Sustainable Aquafeeds: Microalgal (Nannochloropsis sp. QH25) Co-Product Biomass Can Fully Replace Fishmeal in the Feeds for Rainbow Trout (Oncorhynchus mykiss)
Source: Foods. 2025 Feb 25;14(5):781. doi: 10.3390/foods14050781 (PMC11898812; doi:10.3390/foods14050781)
Supplement: Supplementary file 1 [file foods-14-00781-s001.zip › foods-3459062-supplementary.pdf]

Supplementary Tables:

Table S1. Proximate composition and essential amino acids of *Nannochloropsis* sp. QH25 and fishmeal

	Ingredients	
	Nannochloropsis sp. QH25	Fish meal
Proximate composition (%)		
Crude protein	58.03	63.81
Lipid	0.23	11.64
Ash	14.57	0.44
Fiber	1.69	19.29
Energy, kJ g^{-1}	2677.00	3238.00
Essential amino acids(% in the weight of ingredient as is)		
Arginine	2.81	4.57
Lysine	2.66	5.03
Isoleucine	1.60	2.12
Leucine	4.02	3.78
Histidine	0.74	1.37
Methionine	1.06	1.32
Phenylalanine	2.37	2.37
Threonine	2.59	2.73
Tryptophan	0.56	0.23
Valine	2.46	2.72

Table S2. Fatty acid content (% of total fatty acids) defatted *Nannochloropsis* sp. QH25 co-product biomass used in the experimental diets.

Fatty acids (% of TFA)	*Nannochloropsis* sp. QH25 defatted biomass
14:00	0.20
15:00	0.02
16:00	0.87
17:00	0.01
18:00	0.06
20:00	0.01
22:00	0.02
24:00:00	0.02
16:1n-9	0.00
16:1n-7	0.87
18:1n-9	0.22
18:1n-7	0.05
20:1n-9	0.00
20:1n-7	0.00
22:1n-11	0.01
22:1n-9	0.00
24:1n-9	0.00
18:2n-6	0.00
18:3n-6	0.01
20:2n-6	0.01
20:3n-6	0.04
20:4n-6 ARA[1]	0.16
22:4n-6	0.00
22:5n-6	0.03
18:3n-3 ALA[2]	0.01
18:4n-3	0.00
20:3n-3	0.02
20:4n-3	0.00
20:5n-3 EPA[3]	0.56
22:5n-3	0.00
22:6n-3 DHA[4]	0.00
Total SFA[5]	1.25
Total MUFA[6]	1.98
Total n-3 PUFA[7]	0.60

Total n-6 PUFA[8]	0.34
Total PUFA[9]	0.95
Total n-6 LCPUFA[10]	0.23
Total n-3 LCPUFA[11]	0.59

[1]Arachidonic acid (ARA).
[2]Alpha linolenic acid (ALA).
[3]Eicosapentaenoic acid (EPA).
[4]Docosahexaenoic acid (DHA).
[5]Saturated fatty acids (SFA) is the sum of all fatty acids without double bonds.
[6]Monounsaturated fatty acids (MFA) is the sum of all fatty acids with a single bond.
[7]Polyunsaturated fatty acids (PUFA) is the sum of all fatty acids with ≥2 double bonds.
[8]Omega-3 (n-3) PUFA (18:3, 18:4, 20:3, 20:4, 20:5, 22:5, 22:6).
[9]n-6 PUFA (sum of all fatty acids with ≥2 double bonds (18:2, 18:3, 20:2, 20:3, 20:4, 22:4, 22:5).
[10]n-6 long-chain (LC) PUFA (20:2, 20:3, 20:4, 22:4, 22:5).
[11]n-3 LCPUFA (20:3, 20:4, 20:5, 22:5, 22:6).

Table S3. Macrominerals and trace elements in the defatted *Nannochloropsis* sp. QH25 co-product biomass.

	Ingredients
	Nannochloropsis sp. QH25
Macro minerals (%)	
Phosphorus	2.65
Calcium	1.61
Magnesium	1.28
Potassium	1.46
Sulfur	1.96
Trace elements (mg kg−1)	
Copper	0.00
Iron	4.67
Manganese	0.34
Selenium	0.00
Zinc	3.85
Boron	0.02
Aluminum	4.97
Molybdenum	0.00
Arsenic	0.00
Mercury	0.01
Lead	0.01

Table S4. Fatty acid (% of total fatty acids) content of the experimental diets.

Diet TFA %	Reference	33NS	66NS	100NS
14:00	6.03	5.96	7.90	7.19
15:00	0.52	0.52	0.67	0.6
16:00	21.86	21.57	25.13	23.48
17:00	0.62	0.62	0.75	0.70
18:00	4.41	4.34	4.81	4.47
20:00	0.38	0.38	0.42	0.41
22:00	0.26	0.26	0.28	0.25
24:00	0.24	0.24	0.26	0.24
Total SFA[1]	34.42	33.97	40.34	37.44
16:1n-9	0.20	0.19	0.22	0.21
16:1n-7	9.68	9.65	13.0	12.4
18:1n-9	13.75	12.88	13.78	12.59
18:1n-7	2.99	2.95	3.6	3.42
20:1n-9	0.78	0.77	0.85	0.83
20:1n-7	0.12	0.14	0.16	0.17
22:1n-11	0.10	0.10	0.11	0.07
22:1n-9	0.19	0.16	0.23	0.14
24:1n-9	0.34	0.33	0.42	0.32
Total MUFA[2]	28.47	27.49	32.79	30.50
18:2n-6	17.73	17.18	19.55	19.08
18:3n-6	0.25	0.21	0.29	0.29
20:2n-6	0.23	0.23	0.28	0.25
20:3n-6	0.19	0.17	0.24	0.20

20:4n-6 ARA[3]	0.85	0.85	1.17	1.11
22:4n-6	0.00	0.00	0.00	0.00
22:5n-6	0.16	0.15	0.20	0.19
Total n-6 PUFA[4]	0.30	0.30	0.40	0.37
18:3n-3 ALA[5]	19.71	19.10	22.13	21.49
18:4n-3	2.07	1.97	2.58	2.42
20:3n-3	1.48	1.40	2.12	1.94
20:4n-3	0.14	0.13	0.19	0.16
20:5n-3 EPA[6]	0.80	0.79	1.10	1.04
22:5n-3	8.43	8.17	12.08	11.29
22:6n-3 DHA[7]	1.64	1.61	2.23	2.11
Total n-3 PUFA[8]	6.79	6.53	9.42	8.79
Total PUFA	21.73	20.96	30.23	28.22
Total n-6 LCPUFA[9]	44.24	42.67	56.35	53.39
Total n-3 LCPUFA[10]	1.73	1.70	2.29	2.12
n-3/n-6 PUFA ratio[11]	18.18	17.59	25.54	23.86
n-3/n-6 LCPUFA ratio[12]	1.10	1.10	1.37	1.31
20:5n-3 EPA/20:4n-6 ARA	10.51	10.32	11.15	11.25

[1]Saturated fatty acids (SFA) is the sum of all fatty acids without double bonds.
[2]Monounsaturated fatty acids (MUFA) is the sum of all fatty acids with a single bond.
[3]Arachidonic acid (ARA).
[4]Omega-6 (n-6) Polyunsaturated fatty acids (PUFAs) (sum of all fatty acids with ≥2 double bonds (18:2, 18:3, 20:2, 20:3, 20:4, 22:4, 22:5).
[5]Alpha-linolenic acid (ALA).
[6]Eicosapentaenoic acid (EPA).
[7]Docosahexaenoic acid (DHA).
[8]Omega-3 (n-3) PUFAs (18:3, 18:4, 20:3, 20:4, 20:5, 22:5, 22:6).
[9]n-6 long-chain (LC) PUFA (20:2, 20:3, 20:4, 22:4, 22:5).
[10]n-3 LCPUFA(20:3, 20:4, 20:5, 22:5, 22:6).
[11]Ratio calculated for total n-3 PUFA: total n-6 PUFA (n-3/n-6).
[12]Ratio calculated for total n-3 LCPUFA: total n-6 LCPUFA (n-3/n-6).

Table S5. Macrominerals and trace elements in the experimental diets.

	Diet			
	Reference	33N[b]	66N[c]	100N[d]
Macro minerals (%)				
Phosphorus	1.82	1.56	1.43	1.27
Calcium	2.58	1.82	1.24	0.99
Magnesium	0.23	0.28	0.31	0.38
Potassium	1.06	1.18	1.22	1.40
Sulfur	1.49	1.46	1.55	1.51
Trace elements (mg kg^{-1})				
Copper	0.03	0.03	0.03	0.03
Iron	0.15	0.52	0.71	1.11
Manganese	0.01	0.03	0.04	0.06
Selenium	ND[1]	ND[1]	ND[1]	ND[1]
Zinc	0.05	0.04	0.03	0.04
Arsenic	ND[1]	ND[1]	ND[1]	ND[1]
Boron	ND[1]	ND[1]	ND[1]	ND[1]
Aluminum	0.05	0.84	1.45	2.04
Mercury	ND[1]	ND[1]	ND[1]	ND[1]
Lead	0.02	0.01	0.01	0.01
Molybdenum	ND[1]	ND[1]	ND[1]	ND[1]

[1] Not detectable (ND) (<0.000 ug/g).

Table S6. Experimental ingredient market prices obtained from Cruz Aquafeed Sustainability Tool (CAST).

Ingredient	Market price [1]	CAST Source
Fish meal (n=30)	1.54 [1.45, 1.63]	Sarker et al. (2020)
Fish oil (n=30)	1.68 [1.64, 1.76]	Sarker et al. (2020)
Co-product meal (raw)	0.44 [0.40, 0.49]	Sarker et al. (2020)
Blood meal (n=9) [2]	0.85 [0.73, 0.93]	USDA (2022a)
Feather meal (n=92) [3]	0.51 [0.46, 0.53]	USDA (2022b)
Corn gluten meal (n=30)	0.62 [0.59, 0.66]	Sarker et al. (2020)
Soy protein concentrate (n=10)[4]	1.30 [1.00, 3.00]	Alibaba (2022)
Wheat gluten meal (n=10)[5]	1.60 [1.28, 2.41]	Alibaba (2022)

[1] Median [and 95% confidence interval] calculated from bootstrap analysis.
[2] Ruminant blood meal or pork blood meal.
[3] Custom report (2010-2019): "Livestock", "Feedstuffs".
[4]Search term: “soy protein concentrate, feed”.
[5]Search term: “wheat gluten meal, feed”.

Table S7. Whole body proximate composition (wet weight basis) of rainbow trout after 64 days on the experimental diets.

	Whole body (g)[1]				ANOVA	
	Reference	33N	66N	100N	F Value	P Value
Moisture	71.77 ± 0.69	65.35 ± 6.89	73.98 ± 1.41	65.30 ± 7.02	0.79	0.51
Protein	55.83 ± 0.61	56.20 ± 0.39	55.94 ± 1.05	56.06 ± 1.58	0.02	0.99
Fat	35.19 ± 0.81	34.66 ± 0.6	34.62 ± 0.73	34.89 ± 1.36	0.08	0.96
Fiber	0.53 ± 0.10	0.53 ± 0.10	0.67 ± 0.22	0.52 ± 0.13	0.27	0.84
Ash	6.42 ± 0.17	6.66 ± 0.18	6.39 ± 0.20	6.16 ± 0.19	1.22	0.34

[1]Mean ± Standard Error (n=4 replicates per diet; pooled whole tissues of 5 fish/replicate).

Table S8. Proximate filet composition (wet weight basis) of rainbow trout fed experimental diets for 64 days.

Proximate composition (%)						
	Filet[1]				ANOVA	
	Reference	33N	66N	100N	F Value	P Value
Protein	75.51 ± 1.28	74.6.55 ± 0.87	73.38 ± 2.29	74.78 ± 0.72	0.38	0.77
Fat	18.14 ± 1.42	19.74 ± 1.1	19.25 ± 1.34	18.70 ± 1.12	0.30	0.82
Fiber	0.49 ± 0.08	0.90 ± 0.32	0.52 ± 0.17	1.03 ± 0.13	1.93	0.18
Ash	5.24 ± 0.05	5.51 ± 0.38	5.03 ± 0.08	5.37 ± 0.22	0.86	0.49

[1]Mean ± Standard Error (n=4 replicates per diet; pooled whole tissues of 5 fish/replicate).
